# An emerging role of inflammasomes in spinal cord injury and spinal cord tumor

**DOI:** 10.3389/fimmu.2023.1119591

**Published:** 2023-03-09

**Authors:** Jiansong Chen, Yiguo Shen, Xiaobo Shao, Weiliang Wu

**Affiliations:** Department of Orthopedic Surgery, Children’s Hospital, Zhejiang University School of Medicine, National Clinical Research Center for Child Health, Hangzhou, China

**Keywords:** spinal cord injury, spinal cord tumor, inflammasome, caspase-1, immune response

## Abstract

Spinal cord injury (SCI) and spinal cord tumor are devastating events causing structural and functional impairment of the spinal cord and resulting in high morbidity and mortality; these lead to a psychological burden and financial pressure on the patient. These spinal cord damages likely disrupt sensory, motor, and autonomic functions. Unfortunately, the optimal treatment of and spinal cord tumors is limited, and the molecular mechanisms underlying these disorders are unclear. The role of the inflammasome in neuroinflammation in diverse diseases is becoming increasingly important. The inflammasome is an intracellular multiprotein complex and participates in the activation of caspase-1 and the secretion of pro-inflammatory cytokines such as interleukin (IL)-1β and IL-18. The inflammasome in the spinal cord is involved in the stimulation of immune-inflammatory responses through the release of pro-inflammatory cytokines, thereby mediating further spinal cord damage. In this review, we highlight the role of inflammasomes in SCI and spinal cord tumors. Targeting inflammasomes is a promising therapeutic strategy for the treatment of SCI and spinal cord tumors.

## 1 Introduction

Spinal cord injury (SCI) is a devastating event that results in the structural and functional impairment of the spinal cord and may be caused by trauma to or infection or degeneration of the spinal cord (1). SCI incidence is approximately 13 per 100,000 people (2). It results in a varying extent of disability, scoping from partial or complete sensory or motor dysfunction to acute and chronic complications. These complications are accompanied by neuropathic pain, cardiovascular complexities, impaired pulmonary function, pressure ulcers, autonomic dysreflexia, or reduced mobility (3, 4), which have a considerable impact on patients and are an important cause of death after SCI (5). Current treatments for SCI include surgery, drug therapy, and cell therapy. However, these strategies can only improve symptoms and mitigate progression but cannot completely repair the injured spinal cord (6). Thus, SCI can impose a huge psychological and financial burden on individuals as well as society. Taken together, it is necessary to understand the mechanism of SCI and seek an emerging therapeutic blueprint.

The pathological process after SCI can be mainly divided into primary injury and secondary injury. Primary spinal cord injury is caused by the physical injury itself (7), followed by secondary injury caused by a series of biological events, including inflammation, ischemia, oxidative stress, axonal degeneration, astrocyte proliferation, necrosis, apoptosis, and glial scar formation (8). Although the central nervous system (CNS) possesses an innate regenerative capacity, the spinal cord has a poor regenerative ability, which is further complicated by both primary and secondary injuries during SCI. Moreover, the spinal cord regeneration of axons is usually decided by many factors, including the intrinsic growth potential of the CNS neurons, the inhibitory signals produced from CNS myelin damage, reactive astrogliosis, nerve growth factor, and neurotrophic factor (9).

Spinal cord tumors are a heterogeneous group of neoplasms that can be classified into primary and metastatic tumors. Primary spinal tumors account for only approximately 5%–12% of all primary CNS tumors (10) and can be classified into intradural, intramedullary, and extramedullary tumors based on their location. The spinal cord and spine are the common sites of tumor metastasis, and symptomatic metastatic epidural spinal cord compression can occur in 5% to 10% of patients with cancer (11). Owing to the anatomical site of the midline structure, the swelling caused by neuroinflammation is often not tolerated and may result in neurological deficits in both primary and metastatic tumors (12).

SCI and spinal cord tumors are not independent, as spinal tumor-associated compression causes 10% of new-onset SCI and 26% of non-traumatic SCI (13). Patients with spinal tumors might present with acute worsening of neurological function, which often necessitates prompt surgical decompression. Spinal tumors causing SCI are particularly challenging to treat; early diagnosis, multidisciplinary care, and appropriate rehabilitation are necessary to improve the outcomes and quality of life of these patients (14).

The biological process of an immune response is pivotal to the progression and recovery of SCI and the development of spinal cord tumors. Inflammasomes, as innate immune sensors, are multiprotein complexes that play an essential role in defense against pathogens and sterile inflammation (15). Inflammasomes have been demonstrated to be responsible for metabolic diseases, cardiovascular diseases, and tumors (16). However, the mechanism of immune response in SCI and spinal cord tumors remains unclear. This article reviews the mechanism, immune response, and application of inflammasomes in SCI and spinal cord tumors to provide novel insights for the treatment.

## 2 Inflammasome

The inflammasome is a multi-molecular complex consisting of three units: a sensor, an adaptor molecule called ASC (apoptosis-associated speck-like protein containing a caspase-activation and recruitment domain [CARD]), also known as PYCARD, and pro-caspase-1. Currently, the sensors that have been identified include NLR family pyrin domain containing (NLRP)1, NLRP3, and NOD-like receptor family CARD domain containing 4 (NLRC4)), absent in melanoma 2 (AIM2) or pyrin (17, 18). In line with the activation of caspases during the formation of the inflammasome, the inflammasome can be categorized into two types: classical and non-classical inflammasomes, depending on whether or not the inflammation is mediated by the caspase-1 activation (19). Inflammasome activation is usually triggered by host recognition of danger-related molecular patterns (DAMPs) or pathogen-related molecular patterns (PAMPs), which are ligands for the pattern recognition receptors (PRRs) (20). Before inflammasome activation, the PAMPs/DAMPs signals first “trigger” the innate immune cells, transcriptionally upregulating interleukin (IL)-1β and inflammasome sensor expression. When the triggered cells are stimulated by additional PAMPs/DAMPs, the inflammasome complex assembles and initiates a proteolytic cascade, resulting in the hydrolysis and release of IL-1β and IL-18. Programmed cell death in inflammatory forms, called pyroptosis, usually occurs.

In the CNS, DAMPs and PAMPs are mainly expressed by macrophages, astrocytes, and microglia (21). Recognition of DAMPs and PAMPs can induce the transcription and assembly of inflammasome proteins, such that the precursor of caspase-1 is transformed into active caspase-1 *via* autocatalysis. Activated caspase-1 regulates the maturation as well as the release of IL-1β, IL-18, and IL-33 (22). Functionally, IL-1β and IL-18 bind to the corresponding receptor to exert their biological effects (23, 24), whereas IL-33 can promote the T-helper 2 (Th2) to release IL-13 and IL-5 (25). In non-classical activation of inflammasomes, the release of IL-1β is mainly mediated by caspases-4 and 5 (26). Consequently, the secretion of these cytokines further induces the cleavage of gasdermin D (GSDMD) and drives the cell toward pyroptosis (27). Alterations of inflammasome-related pathways have been associated with the development and progression of common immune-mediated and neurodegenerative diseases (28), such as Alzheimer’s disease (29), multiple sclerosis (30), chronic brain injury (31), stroke (31), epilepsy (32), Parkinson disease (33), spinal cord diseases (34), and amyotrophic lateral sclerosis (35).

## 3 Inflammasomes are involved in inflammation and immune response after SCI

**Figure 1:**
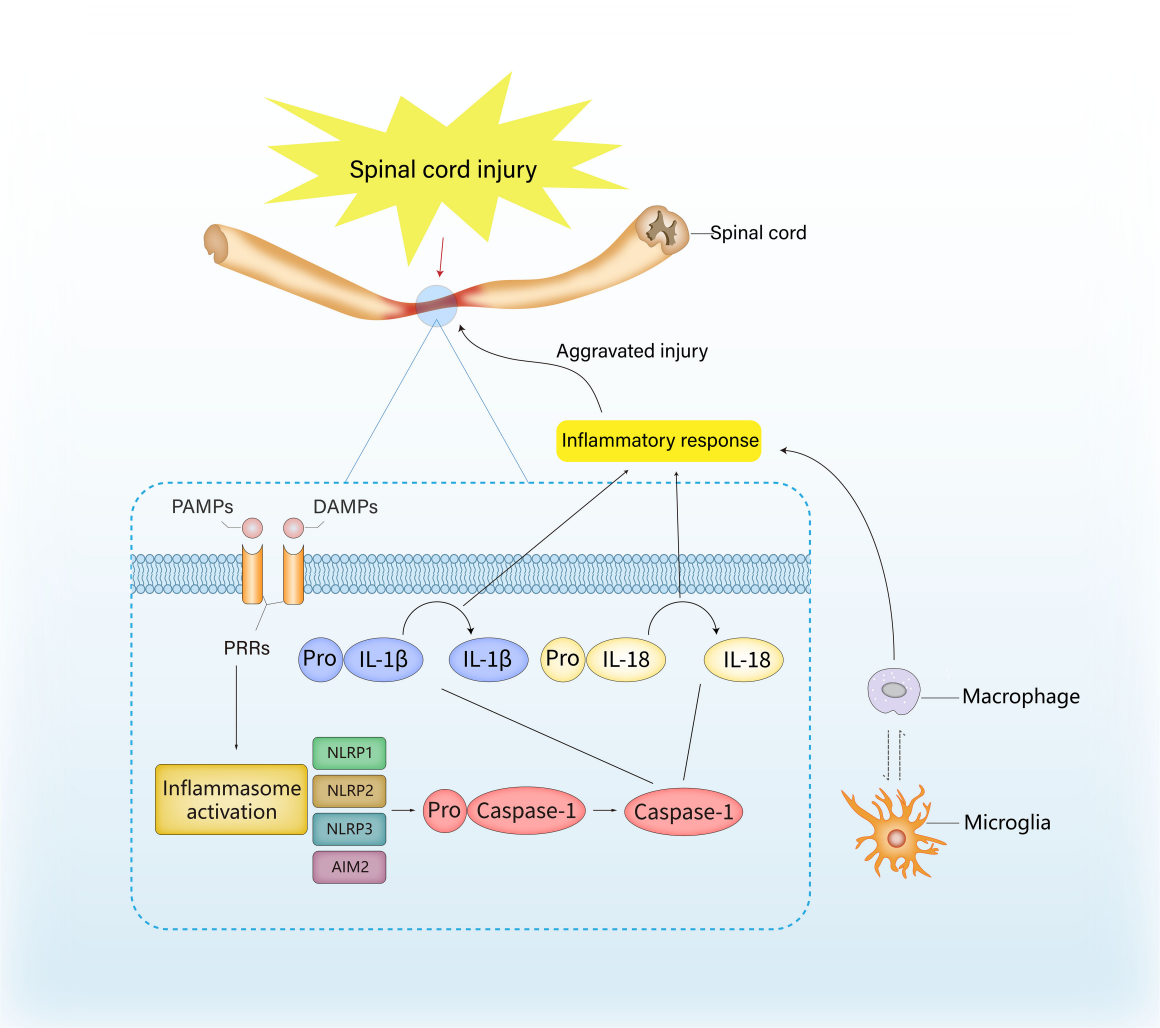
The role of inflammasome in inflammation and immune response after spinal cord injury. The activation of inflammasome is usually launched by host recognition of danger-related molecular patterns (DAMPs) or pathogen-related molecular pattern (PAMPs), which are considered as ligands for the pattern recognition receptors (PRRs). The recognition of DAMPs and PAMPs can induce the transcription and assembly of inflammasome genes, and the precursor of caspase-1 is transformed into active caspase-1 *via* autocatalysis, thereby resulting in the hydrolysis and release of IL-1β and IL-18, ultimately exacerbating the inflammatory response.

SCI can disrupt the homeostasis of the spinal cord microenvironment and result in a series of pathophysiological alterations. Inflammasomes play an essential role in this microenvironmental imbalance, including a shift in the components or their targets, regulation of immune cells, and the secretion of inflammatory factors, thereby impairing regeneration and functional recovery (**Figure 1**).

### 3.1 The role of inflammasomes

#### 3.1.1 NLRP1 inflammasome

As the first inflammasome in the NLR family to be identified in detail, the NLRP1 inflammasome comprises ASC, NLRP1, an inhibitor of apoptosis protein called X-linked inhibitor of apoptosis protein (XIAP), caspase-1, and caspase-11 (36). Structurally, the human NLRP1 protein contains the following protein domains: a CARD, a function to find domain (FIIND), a nucleotide-binding domain (NBD), a leucine-rich repeat (LRR) domain, and an N-terminal PYD (37). NLRP1 inflammasomes have been found in microglia and motor neurons of the spinal cord (17, 38). Significantly enhanced NLRP1 and ASC immunoreactivity has been reported 6 h after moderate experimental cervical SCI, suggesting that SCI can mediate the upregulation of NLRP1 inflammasome (36). Moreover, co-immunoprecipitation of spinal cord lysates and preimmunized serum also revealed overexpression of ASC, NLRP1, and caspase-1 at 24 h after SCI. Interestingly, the cleavage of XIAP into small fragments produces N-terminal baculovirus inhibitor of apoptosis repeat (BIR)1 and BIR2 fragments, which reduce the threshold for caspase-1 activation, resulting in the secretion of IL-1β and IL-18, and aggravation of SCI (39). Additionally, XIAP also mediates innate immune signaling in a receptor interaction protein 2 (RIP2)-dependent manner (40). Vaccari et al. also reported that NLRP1, caspase-1, and ASC levels were elevated after SCI (41).

#### 3.1.2 NLRP2 inflammasome

The NLRP2 inflammasome consists of NLRP2, ASC, and caspase-1, and was discovered in human astrocytes (42). The NLRP2 inflammasome in astrocytes can interact with the P2X7 receptor as well as pannexin-1, which is a transmembrane channel-forming glycoprotein (43). The P2X7 receptor allows the transmembrane fluxes of Ca^2+^ and causes cellular death and necrosis in neurodegenerative diseases (44). Pannexin-1 participates in intracellular Ca^2+^ overload during SCI by accelerating extracellular Ca^2+^ influx, thereby promoting apoptosis of spinal cord neurons (45).

#### 3.1.3 NLRP3 inflammasome

NLRP3, an intracellular receptor, is found in neurons, microglia, and astrocytes (46). NLRP3 becomes activated in response to DAMPs and PAMPs and forms an inflammasome complex with ASC and caspase-1, which subsequently induces the activation and secretion of proinflammatory cytokines (47). The synthesis and activation of the NLRP3 inflammasome usually involve two procedures. First, the initiation of original signaling is triggered by the toll-like receptor/nuclear factor (NF)-κB pathway, increasing inflammasome transcription and promoting posttranslational modifications; this enables the regulation of the expression of NLRP3 inflammasome complexes and the precursors of IL-1β as well as IL-18 under inflammatory situation (48). Consistent with this phenomenon, Ni et al. found a high expression of toll-like receptor 4 and an increase in NF-κB DNA-binding activity at 72 h after SCI (49). The second signal exhibits far-ranging responses to various stimuli, involving the assembly and activation of inflammasome as well as the processing of IL (50). Further, asbestos, extracellular ATP, monosodium urate crystals, the bacterial pore-forming toxin nebramycin, and cholesterol crystals are all known as NLRP3 irritants (51). Huang et al. found that extracellular vesicles derived from epidural fat-mesenchymal stem cells improved neurological functional recovery after SCI, partly by inhibiting the activation of NLRP3 inflammasome (52). Hu et al. demonstrated that NLRP3-related inflammation in motor neurons was induced by microglial activation in the motor cortex, which impaired motor function recovery after SCI. Minocycline inhibited microglia activation, thus reducing NLRP3-related inflammation and promoting functional recovery after SCI (53). More recently, an increasing number of chemicals and molecules, such as trehalose (54), cannabinoid receptor-2 (55), zinc (56), melatonin (57), and dopamine (58), have been found to improve functional recovery after SCI by targeting NLRP3 inflammasome directly or indirectly.

#### 3.1.4 AIM2 inflammasome

AIM2, a cytoplasmic double-stranded DNA (dsDNA) sensor, belongs to the hematopoietic interferon-induced nuclear 200 (HIN200) family; it contributes to the downstream signaling of ASC, which is responsive to the presence of bacterial as well as viral DNA (59). In the normal spinal cord, AIM2 is mainly observed in astrocytes, neurons, and oligodendrocytes (60). AIM2 is also found in activated microglia or macrophages and infiltrated leukocytes during SCI. Moreover, AIM2 can distinguish the DNA released from damaged cells, thereby triggering programmed cell death (61).

### 3.2 The role of immune cells: Microglia, macrophages, neutrophils, and astrocytes

In the CNS, microglia are the main resident macrophages, which play an important role in the process of secondary injury after SCI, especially by regulating the release of proinflammatory cytokines and chemokines (62). The number of activated microglia was shown to increase on the first day after SCI, which then continued to increase for 7 days until the number of cells stabilized between 2 and 4 weeks (63). In the early stages of SCI, activated microglia release trophic factors that promote axonal growth and regeneration at the lesion site and play a neuroprotective role by limiting lesion site enlargement (64). However, activated microglia can also induce peripheral circulating macrophages to infiltrate the injury site and express several pro-inflammatory cytokines such as IL-1α, IL-1β, and tumor necrosis factor-alpha (TNF-α) to mediate the inflammatory response (65). Both macrophages and microglia exert polarized capability with two major phenotypes, M1 and M2 (65). M1-like cells are classically activated and resemble activated microglia by exerting proinflammatory and destructive effects and producing several proinflammatory cytokines (65). Conversely, alternatively activated M2-like cells have strong protective effects that can promote tissue remodeling, wound healing, and angiogenesis. The microenvironment after SCI is detrimental to M2 macrophages, and the overexpression of TNF can inhibit the conversion of M1 to M2 (66). The ratio of M1 to M2 reflects the proportional balance in the spinal cord microenvironment; a disequilibrium in this proportion causes the release of proinflammatory cytokines, including IL-1β, IL-6, and TNF-α. The interplay between inflammasomes and microglia or macrophages has been described by several studies. Zendedel et al. found that ASC, the inflammasome adaptor protein, was predominantly expressed in microglia after SCI (46). Hu et al. demonstrated that microglial activation triggered NLRP3-related inflammation in the motor cortex after SCI (53). Liu et al. found that oxidation protein products, which served as biomarkers of oxidative stress-triggered inflammatory response after SCI, participated in NLRP3-mediated pyroptosis (67). AIM2 exerts regulatory effects in microglia, which is associated with the development of autoimmune encephalomyelitis in a mouse model (68), indicating that an interplay between AIM2 and microglia may also exist in SCI.

Neutrophils are one of the first immune cells entering the injured site after SCI (69). Infiltrating neutrophils cause damage to the blood-spinal barrier (BBB) and induce the release of many inflammatory factors, triggering a cascade of inflammatory effects (69). Various studies have shown that neuroprotective molecules can protect spinal cord tissue by inhibiting inflammasome activity, which is accompanied by reduced infiltration of neutrophils. OLT1177, a selective inhibitor of the NLRP3 inflammasome, reduced the infiltration of neutrophils and showed a protective role in SCI (70). Similar effects and mechanisms have also been observed for BAY 11-7082 or A438079 (71), topotecan (72), P2X4 receptors (73), asiatic acid (74), and polyphenols (75).

After SCI onset, astrocytes infiltrate the injured tissue, secrete several inflammatory factors, and promote fibrosis (76). Mi et al. found that silencing heat shock protein family A member 8 reduced SCI-caused damage by blocking astrocyte activation and lowing NLRP3 levels; knockdown of this protein protected astrocytes from oxygen and glucose deprivation/reoxygenation-induced injury *via* the blockade of NF-κB and NLRP3 inflammasome activation (77). ASC-dependent inflammasome formation, especially in resident cells of the spinal cord, including astrocytes, plays a pivotal role in the progression of secondary damage (78). Extracellular vesicles derived from the mesenchymal stem cells have the potential to regulate inflammasome activity after SCI. Moreover, extracellular vesicles stimulate neural progenitor cells and modulate astrocyte activity (79). Oligodendrocyte progenitor cells have significantly higher levels of inflammasome proteins than astrocytes, which may be associated with their high death rates after SCI (80).

### 3.3 The role of pro-inflammatory or anti-inflammatory cytokines

Cytokines, which can be classified as proinflammatory or anti-inflammatory mediators, are involved in neuroinflammation (81). Various cytokines such as IL-1, IL-6, TNF-α, and leukocyte inhibitory factors are associated with alterations in the microenvironment in SCI (82). When in low concentrations, several proinflammatory cytokines display protective effects by inducing the expression of neurotrophics (83); however, at higher concentrations, these cytokines also mediate the overexpression of neurotoxic genes, such as inducible nitric oxide synthase, proinflammatory proteases, and cyclooxygenase 2 (84). Moreover, IL-1 overexpression in the spinal cord facilitates vascular permeability and lymphocyte recruitment. Additionally, IL-6 promotes the infiltration and activation of macrophages and microglia (85). High levels of TNF-α are found in neurons, glial cells, and endothelial cells after SCI (86). TNF-α can enlist neutrophils to the lesions by inducing adhesion molecules such as intercellular adhesion molecule-1 and vascular cell adhesion protein-1 (87). Subsequently, the permeability of endothelial cells is altered, thereby leading to the disorder of the blood-spinal cord barrier (88). Moreover, TNF-α can induce the death of oligodendrocytes and cause demyelination (89). Generally, CNS cells maintain low IL-1β levels in the brain and spinal cord that are regulated by preassembled inflammasomes (90). Recently, it was shown that expression of NLRP3 inflammasome components increased in the spinal cord tissue of the mouse model of amyotrophic lateral sclerosis and induced superoxide dismutase 1-mediated microglial IL-1β (35).

## 4 The role of inflammasomes in spinal cord tumor

Aberrant activation of the inflammasomes and concurrent overexpression of their effector molecules have been observed in several malignancies (91). Inflammation is a hallmark of neurodegenerative diseases and central nervous tumors (92). SCI results in persistent inflammatory changes, which suggests that SCI may be a risk factor for central nervous tumors, especially spinal cord tumors. Therefore, in this review, we also summarized the research achievements regarding the role of inflammasomes in spinal cord tumors.

### 4.1 Tumorigenesis

A common feature of all cancers is their ability to continuously self-proliferate, which is primarily stimulated by inflammation-driven mechanisms (93). The release of proinflammatory cytokines, such as IL-1β and IL-18, may induce cell proliferation in a paracrine and autocrine manner during acute and chronic inflammation (94, 95). NLRP3 inflammasomes have also been reported to inhibit the function of natural killer cells in the control of carcinogenesis and metastasis (96). In addition, the NLRP3 inflammasome exerts a critical effect on tumorigenesis and may provide prognostic markers and promising therapeutic targets in patients with cancer (97). In the CNS, malignant glioma is the most common primary brain tumor with a poor prognosis. The NLRP3 inflammasome in glioma is also constitutively activated in glioblastoma multiforme cells (98). However, the underlying mechanism of the NLRP3 inflammasome in spinal cord tumorigenesis has not been fully elucidated. Further research is needed to understand the properties of the inflammasome and explore its therapeutic potential in spinal cord tumors. In addition to cytokine maturation, another consequence of inflammasome activation is the cleavage of GSDMD; the cleaved GSDMD forms membrane pores that lead to cytokine release and culminate in cell lysis (99). The exact relevance and function of pyroptosis executor GSDMD during tumorigenesis remain unclear. How these different signals are distributed in different tumor environments and ultimately integrated into different cell types requires further investigation (100).

### 4.2 Metastasis and angiogenesis

Tumor metastasis is an elusive process involving a series of successive events, from the spread of tumor cells from the primary lesion to the development of metastatic foci in distant organs (101). The environment of the distant metastatic target organs experiences reprogramming, primarily through the recruitment of immune cells, in favor of tumor growth. Notably, NLRP3 promotes epithelial–mesenchymal transition by enhancing transforming growth factor-β1 (TGF-β1) signaling and activating small mother against decapentaplegic (SMAD) (102, 103). Angiogenesis, a gradual process of the formation of new capillaries and blood vessels arising from pre-existing vascularity, is necessary for tumor progression (104). It is tightly modulated by multiple pro- and anti-angiogenic factors. Inflammasome complexes contribute to the regulation of angiogenesis in different tissues. IL-1β, produced by tumor cells, induces pro-angiogenic factors. IL-1β mediates the upregulation of hypoxia-inducing factor-1α to stimulate the overexpression of vascular endothelial growth factor (105). Notwithstanding these studies demonstrating the angiogenic function of IL-1β, further exploration is necessary to distinguish how different inflammasome complexes modulate the IIL-1β to regulate angiogenesis in tumors.

### 4.3 Immunosuppression

In response to the invading tumor cells, the immune system initiates a powerful anti-tumor reaction, and inflammatory cells flood the tumor microenvironment (TME) (106). However, cancer cells use several mechanisms to evade the immune system’s surveillance. The release of IL-1β as well as IL-18, is a universally acknowledged process for the immunosuppressive TME during the development of multiple tumors (107, 108). Bone marrow-derived suppressor cells (MDSCs) are crucial components of TME and demonstrate robust immunosuppressive activity (109). NLRP3 is crucial for accumulating MDSCs in tumors and inhibiting the anti-tumor effect of T cells. Moreover, NLRP1 inflammasome promotes the secretion of IL-18 in myeloma, thus resulting in accelerated progression (110).

In summary, the occurrence of tumor is the result of multiple factors, and long-term exposure to the inflammatory microenvironment will increases the risk of tumor development. As an important component of inflammatory response, inflammasome also involve in the occurrence and development of spinal cord tumors, as well as induce the formation of tumor blood vessels, thereby involving in metastasis. Furthermore, inflammasome may prompt the immunosuppression of TME during the development of spinal cord tumors. Although the role of inflammasome in tumors has received considerable attention, studies in spinal cord tumors are still rare. Therefore, further researches focus on the underlying mechanism of inflammasome in spinal cord tumor are quite necessary.

## 5 Inhibiting the inflammasome pathways

As mentioned above, the inflammasomes exert a pivotal effect on SCI. Although there are currently no approved inflammasome suppressor drugs for the treatment of SCI, many therapies are in development and show great promise (**Table 1**).

**Table 1:** Potential therapeutic agents on inflammasome after spinal cord injury.

Targets	Therapeutic agents	Outcome	Mechanism	Does and/or time points of treatment	Experimental animal	Reference
NLRP3 inflammasome inhibition	Methylene blue	Partially inhibit neuronal apoptosis and improve motor function.	Inhibit the protein levels of IL-1β, IL-18 and NLRP3 inflammasome associated with down-regulation of intracellular reactive oxygen species, decreased leukocyte infiltration.	2 mg or 4 mg/kg body weight, 15 minutes before SCI and 3 hours after SCI	Sprague-Dawleyrat	(111)
	Polydatin	Relieve microglial inflammation.	Inhibit iNOS and NLRP3 inflammasome.	20 or 40 mg/kg body weight, 30 minutes after the SCI	Sprague-Dawley rat	(112)
	Wogonoside	Alleviate neuroinflammation.	Alleviate NF-κB and NLRP3 overexpression and increase the activation of IκB.	12, 25 or 50 mg/kg for 10 days	Sprague-Dawleyrat	(113)
	Echinoside	Reduce neuron loss and improve spinal cord structure.	Reduce ROS level, improve the mitochondrial membrane potential, block activation of NF-κB, and inhibit the NLRP3 inflammasome signaling pathway.	20 mg/kg daily until sacrifice	Sprague-Dawleyrat	(114)
	Glycyrrhizin	Functional improvement.	Inhibit NLRP3 inflammasome and promote microglial M2 polarization.	10 g glycyrrhizin given immediately after SCI and every 12 h for 3 days	Sprague-Dawleyrat	(115)
	Paeonol	Promote the recovery of motor function and spinal cord structure, reduce spinal cord edema.	Reduce the levels of ASC, NLRP3, N-GSDMD, repress the contents of IL-1β, IL-18, TNF-α and malondialdehyde, and elevate GSH level.	60 mg/kg daily until sacrifice	Sprague Dawley rat	(116)
	Ulinastatin	Relieve spinal cord edema, ameliorate neurological function and architecture.	Inhibit NLRP3 inflammasome.	50,000 U/kg daily	Sprague-Dawley rat	(117)
	MCC950	Alleviate neuroinflammation.	Inhibit NLRP3 inflammasome.	–	–	(118, 119)
	3,4-methylenedioxy-β-nitrostyrene	Alleviate neuroinflammation.	Inhibit NLRP3 inflammasome.	–	–	(120)
	OLT1177	Inhibit neuroinflammation and improve function	Inhibit NLRP3 inflammasome, reduce IL-1β and IL-18 release.	–	–	(121)
Cytokine inhibition	Adalimumab, infliximab, etanercept	Anti-inflammatory and anti-bacterial effects and promotes recovery after SCI	Inhibit the TNF signaling pathways	–	–	(122)
	Curcumin	neuronal regeneration	inhibiting the expression of NF-κB and TGF-β-SOX9	–	–	(123)
Caspase-1 inhibition	VX-740	Inhibit inflammatory response.	Inhibit caspases-1	–	–	(124, 125)
	VX-765	Inhibit inflammatory response.	Inhibit caspases-1	100 mg/kg, immediately after SCI and continued once daily for 7 days	C57BL/6 mice	(124–126)

### 5.1 NLRP3 inflammasome inhibition

Methylene blue alleviates neuroinflammation post-SCI by inhibiting the activation of the NLRP3 inflammasome in microglia (111). Polydatin, a glycoside of resveratrol, can reduce the activation of the NLRP3 inflammasome and then relieve microglial inflammation, which has a neuroprotective effect on SCI (112). Wogonoside has antioxidant, anti-inflammatory, anti-allergic, and anti-tumor properties (113). Echinoside accelerates the recovery of motor function in rats after SCI by inhibiting the NLRP3 inflammasome (114). Oral glycyrrhizin inhibits NLRP3 inflammasome activation and promotes microglial M2 polarization after a traumatic SC (115). Paeonol may alleviate SCI by regulating the NLRP3 inflammasome and pyroptosis, which is a feasible clinical treatment for SCI (116). Ulinastatin significantly improves neurological function after SCI by regulating the AMP-activated protein kinase/NLRP3 inflammasome signaling pathway (117). MCC950, a small-molecule inhibitor of NLRP3, directly interacts with the Walker B motif within the nucleotide-binding NACHT domain in NLRP3, thereby impeding ATP hydrolysis and inhibiting the synthesis of NLRP3 inflammasome (118, 119). Furthermore, 3,4-methylenedioxy-β-nitrostyrene can block NLRP3-mediated ASC spot formation and oligomerization, but not NLRP3 agonist-induced potassium efflux (120). OLT1177, an orally active β-sulfonyl butyryl molecule, suppresses NLRP3 inflammasome activation. Notably, nanomolar concentrations of OLT1177 can inhibit the secretion of IL-1β and IL-18 following classical and atypical activation of NLRP3 inflammasome *in vitro (* 121). Bigford et al. demonstrated that NLRP3 inflammasome was activated in adipose tissue and pancreas in a chronic SCI mouse model (127). Jiang et al. found that topoisomerase 1 inhibition prevented NLRP3 inflammasome activation and pyroptosis to improve recovery after SCI (128). Antioxidants improved peripheral neuropathy in a tumor-bearing mouse model by regulating spinal cord oxidative stress and inflammation (129). Compared with NLRP3, other inflammasomes have been less studied. Vaccari et al. showed that NLRP1 inflammasome proteins presented in the cerebrospinal fluid of patients with SCI and traumatic brain injury (41). Yutaka et al. found an increased expression level of NLRP2 inflammasome in the dorsal root ganglion, which was associated with inflammatory pain hypersensitivity (130).

### 5.2 Cytokine inhibition

Production of proinflammatory cytokines such as IL-1β is a pivotal step in the development and progression of various neurological diseases. TNF-α-stimulated gene 6 has been demonstrated to be a promising immunomodulatory target in neurodegenerative diseases (131). In terms of SCI, IL-1β has been proposed as a therapeutic target. In 2020, Tang et al. found that binding of a secretory leukocyte protease inhibitor to the promoter region of TNF-α and IL-8 inhibited the NF-κB signaling pathway, which exerts anti-inflammatory and anti-bacterial effects and promotes recovery after SCI (132). TNF is another pivotal cytokine released after SCI onset, as increased TNF expression has been demonstrated throughout the acute and chronic stages of SCI in the resident cells of spinal tissue. TNF inhibitors, such as adalimumab, infliximab, and etanercept, promoted functional recovery after SCI (122). Yuan et al. found that curcumin inhibited a novel cytokine signaling pathway (TGF-β-SOX9) and improved recovery after SCI (123).

### 5.3 Caspase-1 inhibition

Previous studies have demonstrated that the absence of caspase-1 can alleviate neuroinflammation and neuronal damage during SCI, which implicates the potential significance of caspase-1 inhibitors as a therapeutic target. Pralnacasan (VX-740) and its analog VX-765 are peptide-like caspase-1 inhibitors that act by covalent modification of the catalytic site of caspase-1, thereby suppressing the activation of caspase-1 and the subsequent cleavage of the precursors of IL-1β and IL-18 (124, 125). Chen et al. demonstrated that VX-765 reduced neuroinflammation in an SCI mouse model by inhibiting caspase-1/IL-1β/IL-18 (126).

## 6 Perspectives

Aberrantly-activated inflammasomes are involved in SCI and the development of spinal cord tumors; this process depends on several factors, such as the expression patterns and effector molecules of inflammasomes and the profile and composition of the spinal cord microenvironment. However, the current research on inflammasomes in SCI and spinal cord tumors is still scarce, with many unresolved questions, including (1) how is the inflammasome activated in SCI and spinal cord tumors?, (2) what are the effects of the other signaling molecules on the inflammasome and what is the significance of their interaction in the development of SCI and spinal cord tumors?, (3) what is the effect of inflammasome activation in different cell types post-SCI and on the progression of spinal cord tumors?, and (4) what are the effects of each inflammasome pathway on host immunity and immunotherapy? The role of inflammasome-related immune response is less studied, which may be partly due to the difficulty in creating an accurate animal model. With the development of current biotechniques, new models like organoids may be applied to investigate the detailed mechanism in SCI and spinal cord tumors. In this review, we highlight the role of inflammasomes and their effector molecules in SCI and spinal cord tumors. Targeting inflammasomes and effector molecules is expected to bring new hope for treating SCI and spinal cord tumors, which needs to be systematically and comprehensively studied.

## Author contributions

JSC search the literature and wrote the draft. WLW conceived and supervised this work. YGS drawn the figure. YGS and XBS critically revised the manuscript. All authors approved the final version.

## Glossary

**Table:**

NLR	nucleotide-binding domain and leucine-rich repeats
AIM2	absent in melanoma 2
ASC	apoptosis-associated speck-like protein containing a CARD
CARD	caspase activation and recruitment domain
PYD	pyrin domain
IFI16	IFN-γ-inducible protein 16
DAMPs	danger-related molecular patterns
PAMPs	pathogen-related molecular patterns
PRRs	pattern recognition receptors
IL-1β	interleukin-1β
Th2	T-helper 2
GSDMD	gasdermin D
XIAP	X-linked inhibitor of apoptosis protein
FIIND	function to find
NBD	nucleotide-binding domain
LRR	leucine-rich repeat
BIR	baculovirus inhibition repeat
RIP2	receptors interaction protein 2
TLR	toll-like receptors
NF	nuclear factor
dsDNA	double-stranded DNA
TNF-α	tumor necrosis factor alpha
LIF	leukocyte inhibitory factor
iNOS	inducible nitric oxide synthase
COX-2	cyclooxygenase 2
ECs	endothelial cells
ICAM-1	intercellular adhesion molecule
VCAM-1	vascular cell adhesion protein
GBM	glioblastoma multiforme
EMT	epithelial–mesenchymal transition
TGF-β1	transforming growth factor-β1
SMAD	small mothers against decapentaplegic
HIF-1α	hypoxia-inducing factor-1α
VEGF	vascular endothelial growth factor
TME	tumor microenvironment
MDSCs	derived suppressor cells.
